# The causes of bacterial bloodstream infections and antimicrobial resistance patterns in children attending a secondary care hospital in Bhaktapur, Nepal, 2017–2022: a retrospective study

**DOI:** 10.1093/jacamr/dlae035

**Published:** 2024-03-12

**Authors:** Raj Kumar Shrestha, Dhruba Shrestha, Ashok Kumar Sah, Ashmita Thapa, Nipun Shrestha, Ganendra Bhakta Raya, Kenshi Furushima, Bhim Gopal Dhoubhadel, Christopher M Parry

**Affiliations:** Department of Research, Siddhi Memorial Hospital, Bhaktapur, Nepal; Department of Research, Siddhi Memorial Hospital, Bhaktapur, Nepal; Department of Research, Siddhi Memorial Hospital, Bhaktapur, Nepal; Department of Research, Siddhi Memorial Hospital, Bhaktapur, Nepal; Department of Research, Siddhi Memorial Hospital, Bhaktapur, Nepal; Department of Research, Siddhi Memorial Hospital, Bhaktapur, Nepal; School of Tropical Medicine and Global Health (TMGH), Nagasaki University, Nagasaki, Japan; School of Tropical Medicine and Global Health (TMGH), Nagasaki University, Nagasaki, Japan; Department of Respiratory Infections, Institute of Tropical Medicine, Nagasaki University, Nagasaki, Japan; School of Tropical Medicine and Global Health (TMGH), Nagasaki University, Nagasaki, Japan; Clinical Sciences and Education, Liverpool School of Tropical Medicine, Liverpool, UK

## Abstract

**Objectives:**

Data on antimicrobial resistance (AMR) among children in Nepal are limited. Here we have characterized the causes of bacterial bloodstream infections (BSIs), antimicrobial resistance patterns and the mechanisms of β-lactamase production in Enterobacterales among children attending outpatient and inpatient departments of a secondary care paediatric hospital in Nepal.

**Methods:**

We retrospectively collected demographic and clinical data of culture-proven bacterial BSIs between January 2017 and December 2022 among children <18 years attending a 50-bedded paediatric hospital. Stored isolates were subcultured for antimicrobial susceptibility testing against commonly used antimicrobials. Enterobacterales displaying non-susceptibility to β-lactams were phenotypically and genotypically investigated for ESBLs, plasmid-mediated AmpC (pAmpC) β-lactamases and carbapenemases.

**Results:**

A total of 377 significant bacteria were isolated from 27 366 blood cultures. Among 91 neonates with a BSI, *Klebsiella pneumoniae* (*n* = 39, 42.4*%*), *Pseudomonas aeruginosa* (*n* = 15, 16.3%) and *Acinetobacter baumannii* complex (*n* = 13, 14.1%) were most common. In the non-neonates, 275/285 (96.5%) infections were community-acquired including *Staphylococcus aureus* (*n* = 89, 32.4%), *Salmonella* Typhi (*n* = 54, 19.6%) and *Streptococcus pneumoniae* (*n* = 32, 11.6%). Among the 98 *S. aureus*, 29 (29.6%) were methicillin-resistant *Staphylococcus aureus*. *K. pneumoniae* and *Escherichia coli* demonstrated non-susceptibility to extended-spectrum cephalosporins and carbapenems in both community and hospital-acquired cases. For *E. coli* and *K. pneumoniae*, *bla*_CTX-M_ (45/46), *bla*_EBC_ (7/10) and *bla*_OXA-48_ (5/6) were common among their respective groups.

**Conclusions:**

We determined significant levels of AMR among children attending a secondary care paediatric hospital with BSI in Nepal. Nationwide surveillance and implementation of antimicrobial stewardship policies are needed to combat the challenge imposed by AMR.

## Introduction

Antimicrobial resistance (AMR) is a leading global cause of mortality. In 2019, an estimated 1.27 million deaths were directly attributed to bacterial AMR infections.^1^ Death rates per 100 000 population were highest in African regions followed by South Asia and one in every five deaths due to AMR infections occurred in children aged under 5 years.^1^ The burden of AMR infections is high in Nepal as in many low- and middle-income countries.^2^ A 23-year retrospective analysis from a tertiary care centre in Kathmandu has shown gradually increasing levels of MDR infections.^3^ In a further study from the same institution, MDR was reported in 643/719 (89.4%) *Escherichia coli*, 488/532 (91.7%) *Klebsiella* spp., 510/520 (98.1%) *Enterobacter* spp. and 314/382 (82.2%) *Acinetobacter* spp.^4^ High levels of non-susceptibility to third-generation cephalosporins, fluoroquinolones and aminoglycosides among *Klebsiella* spp. and *E. coli*, multidrug resistance in *Acinetobacter* spp. and *Pseudomonas aeruginosa*, and methicillin-resistant *Staphylococcus aureus* (MRSA) is frequent in tertiary care centres.^5–10^

Surveillance is a critical component of AMR containment but studies from tertiary care centres may not be representative of the levels of AMR at lower levels of the healthcare system.^11^ Despite the burden of bacterial infection in children and the high level of antibiotic exposure, especially in those under 5 years,^12^ there is a lack of focus on this age group. Insufficient laboratory capacity^11^ and a lack of local epidemiology data encourage clinicians in low- and middle-income countries to prescribe broad-spectrum antimicrobials as empiric therapy potentially amplifying the emergence and spread of AMR infections.

We have studied the bacterial causes and antimicrobial resistance patterns in culture-proven bloodstream infections (BSIs) among children <18 years old attending a secondary care paediatric hospital in the Kathmandu Valley. We characterize hospital and community-acquired infections and explore the mechanisms of β-lactamase production among Enterobacterales.

## Materials and methods

### Ethics

The study proposal and protocols were reviewed and approved by the ethical review committee of the Nepal Health Research Council (NHRC) (ERB Protocol Registration No. 414/2021 P). All the work was conducted as a part of Siddhi Memorial Hospital’s (SMH) surveillance activities. Anonymous data, with no personal identifiers, were retrieved retrospectively from hospital records. Thus, the need for written consent was waived for the study by the ethical review committee of NHRC.

### Study design and setting

This is a retrospective study conducted at SMH. SMH is a 50-bedded paediatric and maternal secondary level hospital located at Bhaktapur, Nepal. The hospital serves the local population of Bhaktapur municipality, in the Kathmandu Valley. SMH microbiology laboratory performs internal quality controls and participates in inter-laboratory external laboratory quality control programmes. We studied all children with positive bacterial growth from blood culture during the 6 years from 1 January 2017 to 31 December 2022.

### Sample characteristics and laboratory procedures

Febrile children (<18 years old) attending the inpatient or outpatient facility, or inpatients who become unwell, were investigated with a blood culture at the request of the consulting paediatrician. For inpatients, blood was inoculated into a BD BACTEC^TM^ Peds Plus^TM^ bottle (≥2 to 3 mL in infants and children;  ≥ 1 mL of blood for neonates or early infants). Commercial brain heart infusion broth bottles (HiMedia, India) supplemented with 0.05% sodium polyanetholsulfonate were used for blood culture for patients attending outpatient department (OPD). Each sample was incubated aerobically at 37°C for up to 5 days. If turbidity was noted, the broth was inoculated onto 5% sheep blood agar and MacConkey agar plates. The plates were then incubated at 37°C for 18 to 24 hours. Bacterial identification was determined by Gram’s staining, colony characteristics and in-house biochemical tests. The identification scheme at SMH is described in Table S1 (available as Supplementary data at *JAC-AMR* Online). Antimicrobial susceptibility testing was performed by the Kirby–Bauer disc diffusion method. The organisms were stored at the time of isolation in skim-milk tryptone glucose glycerol medium at −40°C.

### Isolate recovery and antimicrobial susceptibility testing

Bacteria isolated from the blood culture of patients were recovered from storage in the freezer by inoculation onto 5% sheep blood agar and MacConkey agar plates followed by aerobic incubation at 37°C for 18 to 24 hours. The isolates were subcultured further until pure isolated colonies were obtained. For cases in which the same organism was obtained from the same patient more than once within the same admission, only the first isolate was studied.

AST was repeated by Kirby–Bauer disc diffusion method according to CLSI guidelines.^13^ Clinically important antimicrobials commonly considered in our hospital for the treatment of bacterial infections were tested against each pathogen. Mueller–Hinton Agar and the antimicrobial discs used for AST were purchased from Mast Group Ltd (Liverpool, UK). Vancomycin and teicoplanin susceptibility were determined by the interpretation of MIC by Etest (Liofilchem). *E. coli* ATCC 25922 and *Staphylococcus aureus* ATCC 25923 were used for quality control of AST.

The assignment of susceptibility categories (resistant, intermediate and susceptible) was performed as per the CLSI guideline.^13^ Interpretation of tigecycline susceptibility for *E. coli* was based on EUCAST clinical breakpoint.^14^  *Staphylococcus aureus* non-susceptible to cefoxitin was categorized as MRSA. MDR was defined as non-susceptibility to any of the antimicrobials in ≥3 antimicrobial classes.^15^ Non-susceptibility was defined as either resistance or intermediate or susceptible dose-dependent phenotype.

### Determination of ESBL, AmpC β-lactamase and carbapenemase production

*E. coli* and *K. pneumoniae* isolates showing non-susceptibility (intermediate or resistant phenotype) to cefoxitin, any third-generation cephalosporin or carbapenems were investigated by D72C tests (Mast Group Ltd, Liverpool, UK) according to the manufacturer’s instructions to detect ESBL, AmpC β-lactamase and suspected carbapenemase production. Isolates presumptively identified as potential carbapenemase producers by the D72C test were investigated by the modified carbapenem inactivation method (mCIM) for confirmation as per the recommendations of the CLSI guideline.^13^

For the isolates showing β-lactamase production, crude DNA extracted by the heat lysis method was subjected to PCR screening for genotyping. Isolates were investigated for *bla*_CTX-M_,^16^  *bla*_SHV_ (not *K. pneumoniae*) and *bla*_TEM_,^17^ five common carbapenemases (*bla*_OXA-48_, *bla*_NDM_, *bla*_KPC_, *bla*_IMP_ and *bla*_VIM)_,^18^ and six plasmid-mediated AmpC β-lactamase (pAmpC) families (*bla*_MOX_, *bla*_CIT_, *bla*_DHA_, *bla*_ACC_, *bla*_EBC_ and *bla*_FOX_).^19^ Carbapenem non-susceptible isolates were investigated for the presence of all three groups of β-lactamases. DNA samples extracted from the clinical isolates previously characterized to harbour the β-lactamases were used as positive controls and the DNA of ATCC *E. coli* 25922 was used as a negative control. Nuclease-free water was used as a no template control.

### Data acquisition and definitions

Demographic and clinical data of the patients were retrieved from the dataset of the microbiology laboratory and medical records at the hospital. BSI cases were considered hospital-acquired if the sample was collected after 48 hours of hospitalization. If the blood was sampled before 48 hours or the child was not admitted to the hospital, the case was categorized as community-acquired. All the cases from the outpatient department and emergency department were considered community-acquired. CoNS (except for cases where CoNS were obtained from two successive blood cultures with identical AST) and *Bacillus* spp. were considered contaminants. Early-onset neonatal sepsis was defined as onset of clinical signs and symptoms of sepsis within 72 hours from birth. Late-onset neonatal sepsis was defined as sepsis after 72 hours.

### Data analysis

The data were entered into an MS Excel sheet (Microsoft) and were imported to SPSS (SPSS v.22; IBM, New York, USA). Variables are presented in proportions and percentages. A Chi-squared test was used to determine the association of β-lactamase production with fluoroquinolone non-susceptibility. A *P* value of <0.05 was considered for statistical significance. Compliance with the Microbiology Investigation Criteria for Reporting Objectively (MICRO) guideline is in the Supplementary Material (Table S2).^20^

## Results

### Demographics and characteristics of infections

**Figure 1.**
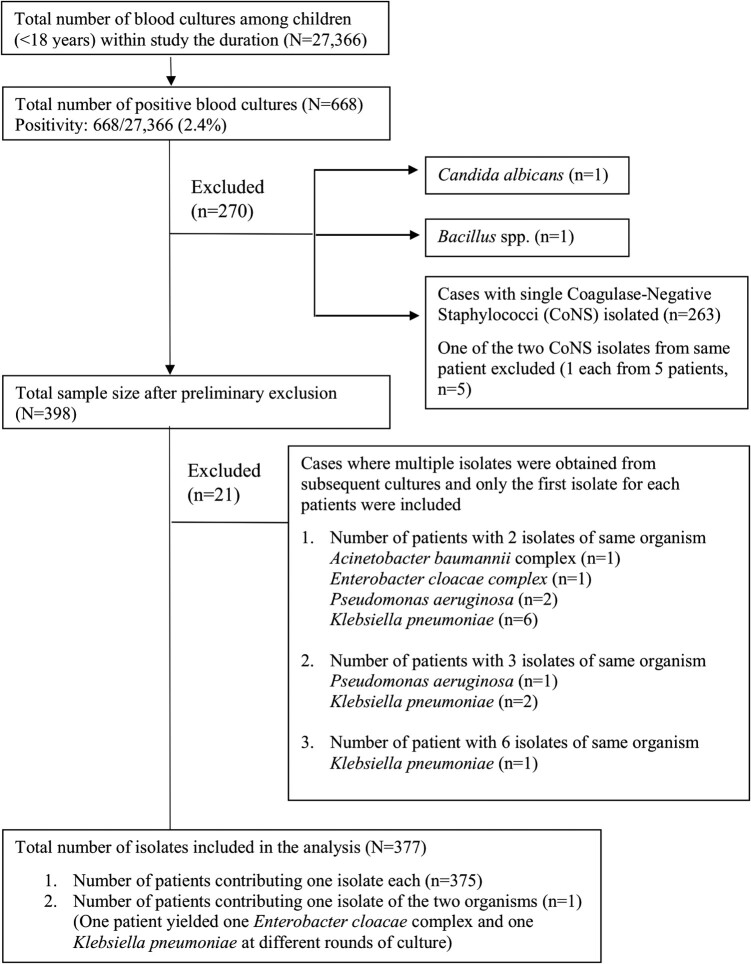
Flowchart showing criteria for selecting isolates included in this study.

A total of 27 366 blood cultures were taken during the 6-year study period. After removing contaminants (coagulase-negative staphylococci and *Bacillus* spp.) and repeated isolates from the same patients, there were a total of 377 significant isolates from 376 patients (Figure 1).

Of the 376 patients, 91 (24.2%) were neonates and 285 (75.8%) non-neonates. Children aged 1 year to <5 years were the most common age group (138/376, 36.7%) (Table 1). Female to male ratio was 71:117. Of 376 children, 146 (38.8%) were hospitalized at SMH.

**Table 1.** Characteristics of 376 children with culture-proven bacterial BSIs

Characteristic	*N* (%)
**Age groups**	
Neonate (0 to <29 days)	91 (24.2)
Infant (29 days to <1 year)	60 (16.0)
Pre-school (1 to <5 years)	138 (36.7)
Children (5 to <13 years)	79 (21.0)
Adolescent (13 to <18 years)	8 (2.1)
**Sex**	
Male	234 (62.2)
Female	142 (37.8)
**Department**	
Outpatient department	167 (44.4)
Paediatric emergency	63 (16.8)
NICU	76 (20.2)
PICU	20 (5.3)
Paediatric ward	34 (9.0)
Cabin	13 (3.5)
Surgery	3 (0.8)
**Site of acquisition**	
Community-acquired	303 (80.6)
Hospital-acquired	73 (19.4)
**Outcome^a^**	
Discharged	109 (74.7)
Death	20 (13.7)
Referred	3 (2.1)
Discharges against medical advice	12 (8.2)
Discharged on request	2 (1.4)

^a^For 146 hospitalized children at SMH.

### Bacterial isolates

The bacterial causes of BSI by age group and site of acquisition are in Table 2. Among the 91 neonates with a BSI, 11 were considered early onset, 54 were late onset and for 27 there were insufficient clinical data to be classified. *K. pneumoniae* (39/92, 42.4%) was the leading cause of BSI among neonates, followed by *P. aeruginosa* (15/92, 16.3%), *A. baumannii* complex (13/92, 14.1%) and *S. aureus* (9/92, 9.8%) (Table 2).

**Table 2.** Bacterial pathogens stratified by age group and site of acquisition

	Age groups (*n*, column % for each age group)							
Organism	Neonate (0 to <29 days) (*n* = 92)	Non-neonate (29 days to < 18 years (*n* = 285)	Infant (29 days to <1 year) (*n* = 60)		Pre-school (1 to <5 years) (*n* = 138)		Children (5 to <13 years) (*n* = 79)	Adolescent (13 to <18 years) (*n* = 8)
	Total	Total	CA (*n* = 52)	HA (*n* = 8)	CA (*n* = 136)	HA (*n* = 2)	CA (*n* = 79)	CA (*n* = 8)
*Staphylococcus aureus* (*n* = 98)	9 (9.8)	89 (31.2)	15 (28.9)	—	47 (34.6)	—	23 (29.1)	4 (50.0)
*Salmonella* Typhi (*n* = 54)	—	54 (19.0)	1 (1.9)	—	19 (14.0)	—	33 (41.8)	1 (12.5)
*K. pneumoniae* (*n* = 73)	39 (42.4)	34 (11.9)	8 (15.4)	6 (75.0)	18 (13.2)	—	1 (1.3)	1 (12.5)
*S. pneumoniae* (*n* = 34)	1 (1.1)	33 (11.6)	9 (17.3)	—	18 (13.2)	1 (50.0)	5 (6.3)	—
*E. coli* (*n* = 34)	6 (6.5)	28 (9.8)	6 (11.5)	1 (12.5)	13 (9.6)	—	7 (8.9)	1 (12.5)
*A. baumannii* complex (*n* = 40)	13 (14.1)	27 (9.5)	5 (9.6)	1 (12.5)	14 (10.3)	1 (50.0)	6 (7.6)	—
*S. pyogenes* (*n* = 6)	—	6 (2.1)	2 (3.9)	—	3 (2.2)	—	1 (1.3)	—
*E. cloacae* complex (*n* = 5)	1 (1.1)	4 (1.4)	2 (3.9)	—	2 (1.5)	—	—	—
*Salmonella* Paratyphi A (*n* = 4)	—	4 (1.4)	—	—	2 (1.5)	—	2 (2.5)	—
*Proteus mirabilis* (*n* = 4)	2 (2.2)	2 (0.7)	1 (1.9)	—	—	—	1 (1.3)	—
*Citrobacter freundii* (*n* = 2)	—	2 (0.7)	2 (3.9)	—	—	—	—	—
*Pseudomonas aeruginosa* (*n* = 16)	15 (16.3)	1 (0.4)	—	—	—	—	—	1 (12.5)
Coagulase-negative Staphylococci (*n* = 5)	4 (4.4)	1 (0.4)	1 (1.9)	—	—	—	—	—
*Serratia marscescens* (*n* = 1)	1 (1.1)	—	—	—	—	—	—	—
*Burkholderia cepacia* complex (*n* = 1)	1 (1.1)	—	—	—	—	—	—	—

‘—’ means no observations.

CA, Community-acquired; HA: Hospital-acquired.

In the non-neonates, 275/285 (96.5%) infections were community-acquired including *Staphylococcus aureus* (89/275, 32.4%), *Salmonella* Typhi (54/275, 19.6%), *Streptococcus pneumoniae* (32/275, 11.6%), *Klebsiella pneumoniae* (28/275, 10.2%), *E. coli* (27/275, 9.8%), *Acinetobacter baumannii* complex (25/275, 9.1%), *Streptococcus pyogenes* (6/275, 2.2%), *Salmonella* Paratyphi A (4/275, 1.5%) and *Enterobacter cloacae* complex (4/275, 1.5%). *S. aureus*, *E. coli* and *A. baumannii* complex were common in all age groups, the typhoidal salmonellae occurred in those aged 1 to 13 years, and *S. pneumoniae*, *K. pneumoniae* and *E. cloacae* complex in those under 5 years. *K. pneumoniae* was the main bacteria isolated in the hospital-acquired BSI in this group.

### Antimicrobial resistance

The antimicrobial non-susceptibility patterns are shown in Table 3(a) (neonates) and Table 3(b) (non-neonates), and the non-susceptibility data stratified by the site of acquisition are presented in Table S3(a) (community-acquired) and Table S3(b) (hospital-acquired). In the neonates, there were high levels of extended-spectrum cephalosporin non-susceptibility among *K. pneumoniae* (32/39, 82%) and *E. coli* (3/6, 50%). Non-susceptibility to other antimicrobials was common in *K. pneumoniae* including meropenem in 4/39 (10%), piperacillin-tazobactam in 12/39 (31%), ciprofloxacin in 26/39 (67%) and gentamicin in 12/39 (31%), but not in *E. coli*. In the non-neonates, extended-spectrum cephalosporin non-susceptibility was present in 20/34 (59%) *K. pneumoniae* and 15/28 (54%) *E. coli*. Non-susceptibility to meropenem was 6/34 (18%) in *K. pneumoniae* and 3/28 (11%) in *E. coli*. The proportions of non-susceptibilities ranged from 18% to 26% for gentamicin and piperacillin-tazobactam and 32% to 47% for ciprofloxacin in these two Gram-negative bacteria (GNB). Levels of non-susceptibilities were low for the *A. baumannii* complex and *P. aeruginosa* except for trimethoprim-sulfamethoxazole non-susceptibility in *A. baumannii* complex isolates in 4/13 (31%) neonates and 7/27 (26%) non-neonates. Ciprofloxacin resistance was detected in 21/54 (39%) of the *Salmonella* Typhi isolates and 2/4 (50%) *Salmonella* Paratyphi, but there was no resistance to ceftriaxone or azithromycin. Antimicrobial non-susceptibility was infrequent in the small number of other GNBs.

**Table 3.** Antimicrobial non-susceptibility patterns of bacterial isolates obtained from blood cultures of the three hundred and 76 paediatric patients

(a) Neonates (*n* = 92)															
		*K. pneumoniae* (*n* = 39)	*E. coli* (*n* = 6)	*A. baumannii* complex (*n* = 13)	*Pseudomonas aeruginosa* (*n* = 15)	*E. cloacae* complex (*n* = 1)	*Proteus mirabilis* (*n* = 2)	*Burkholderia cepacia complex* (*n* = 1)	*Serratia marcescens* (*n* = 1)		*Staphylococcus aureus* (*n* = 9)	MRSA (*n* = 5)	MSSA (*n* = 4)	*S. pneumoniae* (*n* = 1)	CoNS (*n* = 4)
Antimicrobial non-susceptibility, No. of non-susceptible isolates/No. of total isolates tested (%)	**AP**	—	5/6 (83)	—	—	—	—	—	—	**PG**	7/9 (78)	5/5 (100)	2/4 (50)	0/1 (0)	0/4 (0)
	**AUG**	22/39 (56)	2/6 (33)	—	—	—	—	—	—	**FOX**	5/9 (56)	5/5 (100)	0/4 (0)	—	—
	**PTZ**	12/39 (31)	0/6 (0)	1/13 (8)	0/15 (0)	0/1 (0)	0/2 (0)	0/1 (0)	0/1 (0)	**CPD**	5/9 (56)	5/5 (100)	0/4 (0)	—	—
	**SAM**	—	—	1/13 (8)	—	—	—	—	—	**E**	7/9 (78)	4/5 (80)	3/4 (75)	0/1 (0)	2/4 (50)
	**CTX**	35/39 (90)	3/6 (50)	—	—	0/1 (0)	0/2 (0)	—	—	**CD**	1/9 (11)	1/5 (20)	0/4 (0)	0/1 (0)	1/4 (25)
	**CAZ**	32/39 (82)	3/6 (50)	1/13 (8)	2/15 (13)	0/1 (0)	0/2 (0)	1/1 (100)	0/1 (0)	**CIP**	6/9 (67)	4/5 (80)	2/4 (50)	0/1 (0)	0/4 (0)
	**CPM**	19/39 (49)	0/6 (0)	1/13 (8)	2/15 (13)	0/1 (0)	0/2 (0)	0/1 (0)	0/1 (0)	**VAN**	0/9 (0)	0/5 (0)	0/4 (0)	0/1 (0)	0/4 (0)
	**IMI**	—	—	1/13 (8)	0/15 (0)	0/1 (0)	0/2 (0)	0/1 (0)	0/1 (0)	**TEI**	0/5 (0)	0/3 (0)	0/2 (0)	—	0/4 (0)
	**MEM**	4/39 (10)	0/6 (0)	1/13 (8)	0/15 (0)	0/1 (0)	0/2 (0)	0/1 (0)	0/1 (0)	**GM**	1/9 (11)	1/5 (20)	0/4 (0)	0/1 (0)	0/4 (0)
	**CIP**	26/39 (67)	1/6 (17)	1/13 (8)	6/15 (40)	0/1 (0)	0/2 (0)	1/1 (100)	0/1 (0)	**DXT**	0/5 (0)	0/3 (0)	0/2 (0)	—	0/4 (0)
	**AK**	8/39 (21)	0/6 (0)	0/13 (0)	4/15 (27)	0/1 (0)	0/2 (0)	0/1 (0)	0/1 (0)	**C**	0/5 (0)	0/3 (0)	0/2 (0)	0/1 (0)	0/4 (0)
	**GM**	12/39 (31)	0/6 (0)	0/13 (0)	—	0/1 (0)	0/2 (0)	0/1 (0)	0/1 (0)	**LZD**	0/5 (0)	0/3 (0)	0/2 (0)	—	0/4 (0)
	**TGC**	—	0/6 (0)	—	—	—	—	—	—	**TS**	2/9 (22)	1/5 (20)	1/4 (25)	1/1 (100)	1/4 (25)
	**TS**	18/39 (46)	4/6 (67)	4/13 (31)	—	0/1 (0)	0/2 (0)	0/1 (0)	0/1 (0)	**LEV**	—	—	—	—	—

(b) Non-neonates (*n* = 285)																		
		*K. pneumoniae* (*n* = 34)	*E. coli* (*n* = 28)	*A. baumannii* complex (*n* = 27)	*Pseudomonas aeruginosa* (*n* = 1)	*E. cloacae* complex (*n* = 4)	*Proteus mirabilis* (*n* = 2)	*Citrobacter freundii* (*n* = 2)		*Salmonella* Typhi (*n* = 54)	*Salmonella* Paratyphi A (*n* = 4)		*Staphylococcus aureus* (*n* = 89)	MRSA (*n* = 24)	MSSA (*n* = 65)	*S. pneumoniae* (*n* = 33)	*S. pyogenes* (*n* = 6)	CoNS (*n* = 1)
Antimicrobial non-susceptibility, No. of non-susceptible isolates/No. of total isolates tested (%)	**AP**	—	15/28 (54)	—	—	—	—	—	**AP**	2/54 (4)	0/4 (0)	**PG**	53/89 (60)	24/24 (100)	29/65 (45)	1/33 (3)	0/6 (0)	0/1 (0)
	**AUG**	15/34 (44)	8/28 (29)	—	—	—	—	—	**C**	0/54 (0)	0/4 (0)	**FOX**	24/89 (27)	24/24 (100)	0/65 (0)	—	—	—
	**PTZ**	9/34 (26)	5/28 (18)	1/27 (4)	0/1 (0)	0/4 (0)	0/2 (0)	0/2 (0)	**TS**	0/54 (0)	0/4 (0)	**CPD**	24/89 (27)	24/24 (100)	0/65 (0)	—	—	—
	**SAM**	—	—	2/27 (7)	—	—	—	—	**PEF**	11/14 (79)	1/1 (100)	**E**	56/89 (63)	17/24 (71)	39/65 (60)	4/33 (12)	0/6 (0)	0/1 (0)
	**CTX**	20/34 (59)	15/28 (54)	—	—	1/4 (25)	0/2 (0)	—	**CIP**	21/54 (39)	2/4 (50)	**CD**	18/89 (20)	6/24 (25)	12/65 (18)	0/33 (0)	0/6 (0)	0/1 (0)
	**CAZ**	20/34 (59)	14/28 (50)	2/27 (7)	0/1 (0)	1/4 (25)	0/2 (0)	0/2 (0)	**CRO**	0/54 (0)	0/4 (0)	**CIP**	29/89 (33)	16/24 (67)	13/65 (20)	1/33 (3)	0/6 (0)	0/1 (0)
	**CPM**	13/34 (38)	10/28 (36)	1/27 (4)	0/1 (0)	0/4 (0)	0/2 (0)	0/2 (0)	**ATH**	0/54 (0)	0/4 (0)	**VAN**	0/89 (0)	0/24 (0)	0/65 (0)	0/33 (0)	0/6 (0)	0/1 (0)
	**IMI**	—	—	0/27 (0)	0/1 (0)	0/4 (0)	0/2 (0)	0/2 (0)	**MEM**	0/54 (0)	0/4 (0)	**TEI**	0/33 (0)	0/13 (0)	0/20 (0)	—	—	0/1 (0)
	**MEM**	6/34 (18)	3/28 (11)	0/27 (0)	0/1 (0)	0/4 (0)	0/2 (0)	0/2 (0)	**GM**	0/54 (0)	0/4 (0)	**GM**	7/89 (8)	3/24 (13)	4/65 (6)	2/33 (6)	0/6 (0)	0/1 (0)
	**CIP**	16/34 (47)	9/28 (32)	1/27 (4)	0/1 (0)	0/4 (0)	1/2 (50)	0/2 (0)				**DXT**	0/33 (0)	0/13 (0)	0/20 (0)	0/5 (0)	—	0/1 (0)
	**AK**	6/34 (18)	5/28 (18)	0/27 (0)	0/1 (0)	0/4 (0)	0/2 (0)	0/2 (0)				**C**	2/36 (6)	2/14 (14)	0/22 (0)	0/33 (0)	0/6 (0)	0/1 (0)
	**GM**	8/34 (24)	7/28 (25)	0/27 (0)	—	0/4 (0)	0/2 (0)	0/2 (0)				**LZD**	0/33 (0)	0/13 (0)	0/20 (0)	—	—	0/1 (0)
	**TGC**	—	0/28 (0)	—	—	—	—	—				**TS**	25/89 (28)	8/24 (33)	17/65 (26)	9/33 (27)	2/6 (33)	0/1 (0)
	**TS**	11/34 (32)	6/28 (21)	7/27 (26)	—	1/4 (25)	0/2 (0)	0/2 (0)				**LEV**	—	—	—	0/33 (0)	0/6 (0)	—

‘—’ means not-tested.

AP, ampicillin; AUG, amoxicillin-clavulanic acid; PTZ, piperacillin-tazobactam; SAM, ampicillin-sulbactam; CTX, cefotaxime; CAZ, ceftazidime; CPM, cefepime; IMI, imipenem; MEM, meropenem; CIP, ciprofloxacin; AK, amikacin; GM, gentamicin; TGC, tigecycline; TS, trimethoprim-sulfamethoxazole; C, chloramphenicol; PEF, pefloxacin; CRO, ceftriaxone; ATH, azithromycin; PG, penicillin; FOX, cefoxitin; CPD, cefpodoxime; E, erythromycin; CD, clindamycin; VAN, vancomycin; TEI, teicoplannin; DXT, doxycycline; LZD, linezolid; LEV, levofloxacin; MSSA, methicillin-susceptible *Staphylococcus aureus*.

In non-neonates, 24/89 (27.0%) of the *S. aureus* isolates were MRSA, and many of these isolates were also resistant to erythromycin, ciprofloxacin and trimethoprim-sulfamethoxazole. Notably, 5/9 (55.6%) of *S. aureus* isolates in the neonates were MRSA. Of the 29 MRSA BSI, 26 were community-acquired and most were in the pre-school age group (13/29, 44.8%). No *S. aureus* was non-susceptible to vancomycin. Additional testing on a sub-set of 38 isolates confirmed susceptibility to vancomycin and teicoplanin [median MIC 0.25 mg/L (IQR 0.19–0.5); teicoplanin, (median MIC 0.5 mg/L (IQR 0.38–1)]. Levels of non-susceptibility to relevant antimicrobials for *S. pneumoniae* and *S. pyogenes* were low.

Overall, 105/377 (27.9%) isolates were MDR. Three bacteria, *K. pneumoniae* (51/105), *S. aureus* (33/105) and *E. coli* (17/105), accounted for 96.2% of all MDR isolates. The proportion of MDR was highest among *K. pneumoniae* (51/73, 69.9%), neonates (41/92, 44.6%), females (45/143, 31.5%) and hospital-acquired infections (32/74, 43.2%).

### Characteristics of β-lactamase producing isolates

Of 18 *E. coli* and 55 *K. pneumoniae* non-susceptible to third-generation cephalosporins and/or cefoxitin, 15 *E. coli* and 41 *K. pneumoniae* were available for further analysis. Among these isolates, 13 *E. coli* and 40 *K. pneumoniae* were found to display at least one phenotypic mechanism of β-lactamase production. ESBL was the most common mechanism of β-lactamase production (*n* = 46) followed by pAmpC β-lactamase (*n* = 10) and carbapenemase (*n* = 6) (Table 4).

**Table 4.** Distribution of ESBL, pAmpC β-lactamase and carbapenemase genotypes among 13 *E. coli* and 40 *K. pneumoniae* isolates

Pathogen	β-lactamase type			*n* (%)	Neonates *n* (% of 28)	Non-neonates *n* (% of 25)
	ESBL	pAmpC	Carbapenemase			
*E. coli*	*bla*_CTX-M_			8 (15.1)	2 (7.1)	6 (24.0)
		*bla*_EBC_		1 (1.9)	—	1 (4.0)
	*bla*_TEM_	*bla*_DHA_		1 (1.9)	—	1 (4.0)
	*bla*_CTX-M_ *bla*_SHV_	*bla*_EBC_		1 (1.9)	—	1 (4.0)
	*bla*_CTX-M_		*bla*_NDM_ *bla*_OXA-48_	2 (3.8)	—	2 (8.0)
*K. pneumoniae*	*bla*_CTX-M_			24 (45.3)	14 (50.0)	10 (40.0)
	*bla*_CTX-M_ *bla*_TEM_			5 (9.4)	4 (14.3)	1 (4.0)
			*bla*_NDM_	1 (1.9)	—	1 (4.0)
			*bla*_OXA-48_	1 (1.9)	—	1 (4.0)
		*bla*_DHA_ *bla*_EBC_		1 (1.9)	1 (3.6)	—
		*bla*_EBC_		3 (5.7)	3 (10.7)	—
	*bla*_CTX-M_	*bla*_DHA_		1 (1.9)	1 (3.6)	—
	*bla*_CTX-M_ *bla*_TEM_	*bla*_DHA_		1 (1.9)	1 (3.6)	—
	*bla*_CTX-M_ *bla*_TEM_	*bla*_EBC_		1 (1.9)	—	1 (4.0)
	*bla*_CTX-M_		*bla*_OXA-48_	1 (1.9)	1 (3.6)	—
	*bla*_CTX-M_		*bla*_NDM_ *bla*_OXA-48_	1 (1.9)	1 (3.6)	—

‘—’ means no observation.

Among the β-lactamases investigated, *bla*_CTX-M_ (*n* = 45), *bla*_EBC_ (*n* = 7) and *bla*_OXA-48_ (*n* = 5) were the most frequent ESBL, pAmpC β-lactamase, and carbapenemase among *E. coli* and *K. pneumoniae*, respectively (Table 4). Four and five isolates had the simultaneous presence of ESBL-carbapenemase and ESBL-pAmpC β-lactamase combination, respectively.

Among 45 neonatal BSIs caused either by *E. coli* or *K. pneumoniae*, 20 were ESBL, four were pAmpC β-lactamase, two were ESBL-pAmpC and two were ESBL-carbapenemase producers (Table 4). One carbapenem-resistant *A. baumannii* complex was confirmed to be positive for *bla*_NDM_.

**Figure 2.**
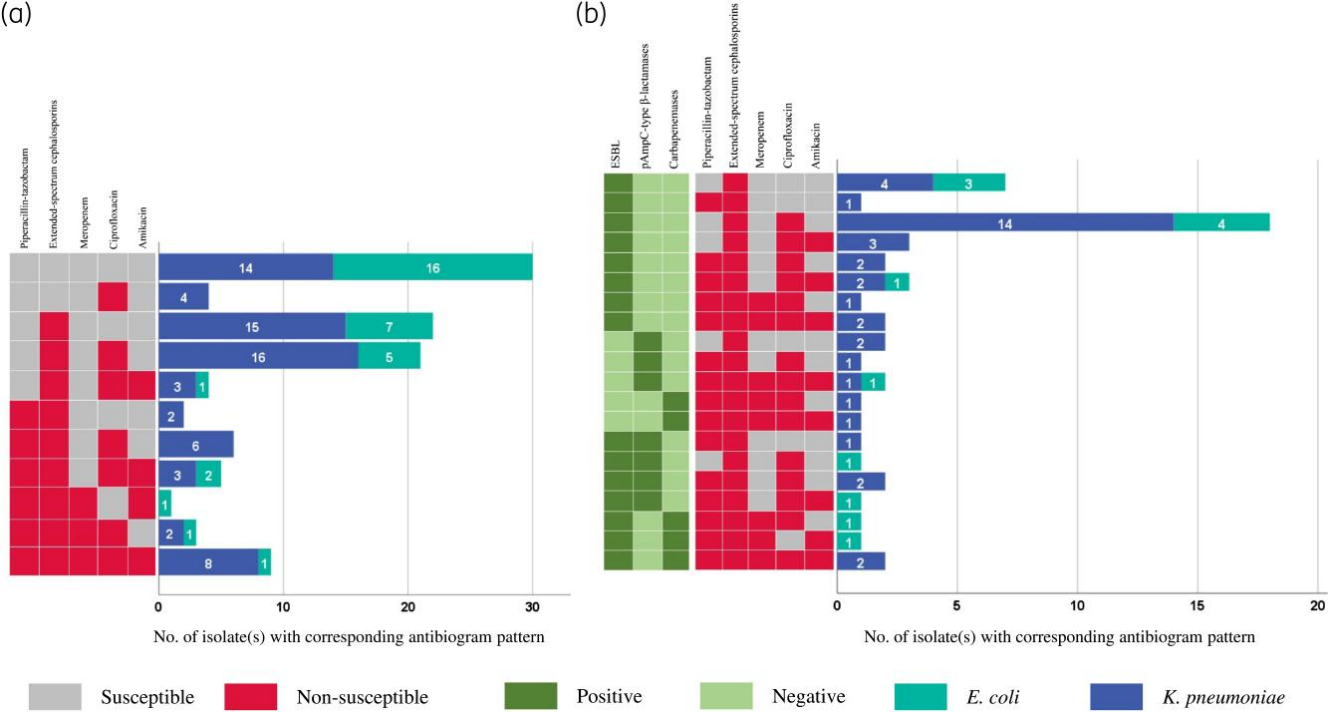
Antibiogram of (a) 73 *K. pneumoniae* and 34 *E. coli* and (b) 53 β-lactamase-producing *E. coli* and *K. pneumoniae* isolates.

Overall, ciprofloxacin non-susceptibility in *E. coli* (10/34, 29.4%) and *K. pneumoniae* (42/73, 57.5%) were significantly associated with β-lactamase production (*χ*^2 ^= 34.8, *P* = 0.000). Consequently, non-susceptibility to ciprofloxacin was common among isolates with non-susceptibility to extended-spectrum cephalosporins (48/73; 65.8%) (Figure 2a). Analysis of β-lactamase producing isolates revealed additional higher proportions of non-susceptibility to gentamicin (22/53; 41.5%) and amikacin (15/53; 28.3%) (Figure 2b).

### Characteristics of fatal BSIs

There were 20 deaths among the 146 patients with positive blood cultures who were hospitalized at SMH, of whom 17 were neonates, two were infants and one was a 12-year-old child. One each of the neonatal deaths was associated with an *A. baumannii* complex isolate harbouring *bla*_NDM_ (newborn had hypoxic-ischaemic encephalopathy), CoNS (newborn had congenital heart disease, CHD) and *E. coli* with no acquired resistance (newborn had moderate to late prematurity). Of nine fatal *K. pneumoniae* BSIs (all ceftazidime resistant), four were ESBL positive, one was pAmpC β-lactamase positive and three had both ESBL and pAmpC β-lactamase. Three of these neonates were premature and had low birth weight (two very pre-term and one moderate to late pre-term). Another six neonatal deaths were associated with *P. aeruginosa*; one had birth asphyxia and five were premature (one moderate to late pre-term and four very pre-term) and of low birth weight. The remaining two deaths were associated with *Serratia marscescens* (neonate with CHD) and MRSA (children aged 12 years).

## Discussion

We report the bacterial causes of BSIs in children attending a secondary healthcare level in the Kathmandu Valley, Nepal. In neonates, GNB, which were probably hospital-associated, were the main cause of BSI. In the non-neonates, most BSIs were community-acquired. Six of the WHO GLASS AMR pathogens (*Acinetobacter* spp., *E. coli*, *K. pneumoniae*, *Salmonella* spp., *S. aureus* and *S. pneumoniae*)^2^ comprised 337/377 (89.4%) of the BSIs. We found a high level of MDR in *K. pneumoniae* (51/73; 69.9%), *E. coli* (17/34; 50.0%) and *S. aureus* (33/98; 33.7%), in agreement with previous findings from Nepal.^3–10^

There were high levels of extended-spectrum cephalosporin resistance among BSIs in this population, particularly among *E. coli* and *K. pneumoniae*, and most of these infections were from children <5 years. These isolates were often also non-susceptible to fluoroquinolones and aminoglycosides.^4,21^ Carbapenem non-susceptibility levels of 3/34 (9%) in *E. coli* and 10/73 (14%) in *K. pneumoniae* were higher than in other settings such as in Cambodia,^22^ Malawi,^23^ but lower than in India.^24^  *Bla*_CTX-M_ was the most common ESBL gene in *E. coli* and *K. pneumoniae*, consistent with findings from surveillances in Asia-Pacific,^25^ Thailand,^21^ Malawi^23^ and India.^26^ Surprisingly, *bla*_EBC_ (7/10) was the most common pAmpC and we did not detect *bla*_CIT_ (such as CMY-2). In another study of clinical isolates from Nepal, the positivity for *bla*_CIT_ was 25/72 (34.7%) and 9/25 (36.0%) for *E. coli* and *K. pneumoniae*, respectively.^27^  *Bla*_DHA_ was present in 25/72 (34.7%) and 10/25 (40.0%) among *E. coli* and *K. pneumoniae*, respectively. We found a small number of carbapenemase-harbouring isolates with *bla*_NDM_ and *bla*_OXA-48_ which are common carbapenemases in Asia,^21,25^ including Nepal.^4^ The rare or low occurrence of *bla*_KPC_, *bla*_IMP_ and *bla*_VIM_ in Nepal is consistent with our observations.^4^ We also demonstrate the co-existence of ESBL-carbapenemase and ESBL-pAmpC β-lactamase combinations in individual isolates. *A. baumannii* complex and *P. aeruginosa* were mostly susceptible to antimicrobials tested with only two *A. baumannii* complex (5.0%) and one *P. aeruginosa* (6.3%) displaying MDR. This is in contrast to high levels of resistance reported in *Acinetobacter* spp. and *P. aeruginosa* in other studies in Nepal.^4,8,9^

*S. aureus* and *S. pneumoniae* were the first and third most common community-acquired BSIs in non-neonates. The proportion of MRSA in this study (29/98; 29.6%) was consistent with a pooled estimate for Nepal, 38.2% (95% CI: 31.4%–45.2%),^6^ and WHO’S GLASS reports’ median MRSA BSIs of 34.7%.^2^ Most MRSA BSI [26/29 (89.7%%)] were thought to be community-acquired, mostly among children aged 1 to <13 years, although it is possible previous hospital contact was not recorded. In view of the nationwide vaccine coverage of pneumococcal conjugate vaccine, it would be instructive to understand the pneumococcal conjugate vaccine status among these children and the serotype of the invasive isolate. *Salmonella* Typhi and Paratyphi A accounted for 58 (19.1%) of community-acquired infections. Ciprofloxacin non-susceptibility (23/58; 39.7%) was lower than reported in other South Asian countries.^11^ The recent introduction of the Vi capsular polysaccharide conjugate vaccine into the routine immunization programme should reduce the future burden of enteric fever among children in Nepal.

There were 20 deaths: 17 in neonates and two were infants. BSIs may have been a contributor rather than a direct cause of death since most children had underlying health conditions, mostly prematurity and low birth weight. Deaths associated with culture-proven BSI as a proportion of the total neonatal intensive care unit (NICU) admitted cases in our institution were low [17 of 961 (1.8%) NICU and 2 of 624 (0.3%) paediatric intensive care unit (PICU) admissions within the study period]. The high proportions of BSI in neonates non-susceptible to ampicillin and gentamicin and to extended-spectrum cephalosporins challenge empiric antimicrobial therapy for neonatal sepsis in this setting.^28^ Carbapenem-sparing options would be welcomed. Piperacillin-tazobactam, colistin and tigecycline could be options, but clinical data in neonates and young children are needed.

This study has limitations. The proportion of positive blood cultures was low, possibly due to the use of in-house blood culture methods for the outpatients. Also, we did not record the volume of blood inoculated in each instance and low blood volumes may have been contributory. We categorized hospital-acquired from community-acquired infections as per the CDC’s recommendations for children hospitalized at SMH, as advised in the MICRO guideline.^20^ In this retrospective study, we were unable to subdivide the community-onset infections in children with known co-morbidities and previous healthcare contact (healthcare-associated) and those in otherwise healthy children (community-acquired). We excluded most Coagulase-negative staphylococci BSIs even though a small proportion of these cases in neonates may have been true pathogens. We did not determine other mechanisms of resistance in extended-spectrum cephalosporin-non-susceptible isolates, such as efflux pump over-expression, porin alterations and the presence of other resistance genes not covered by the assay.

MDR BSI is a significant challenge in the paediatric population in Nepal. *K. pneumoniae*, *E. coli* and *S. aureus* were among the leading BSI-causing pathogens and also demonstrated high levels of MDR. The former two were found to be mostly ESBL producers and our data suggest the need for carbapenem-sparing options for the treatment of ESBL BSIs particularly in neonatal sepsis. Nationwide AMR surveillance, including in secondary and primary care settings, and antimicrobial stewardship initiatives are necessary to combat AMR in Nepal.

## Supplementary Material

dlae035_Supplementary_Data

## Data Availability

All data generated or analysed during this study are included in this published article.

## Supplementary data

Tables S1, S2, and S3 are available as Supplementary data at *JAC-AMR* Online.
